# Facial expression recognition based on improved depthwise separable convolutional network

**DOI:** 10.1007/s11042-022-14066-6

**Published:** 2022-11-23

**Authors:** Hua Huo, YaLi Yu, ZhongHua Liu

**Affiliations:** 1 Engineering Technology Research Center of Big Data and Computational Intelligence, Henan University of Science and Technology, Kaiyuan Avenue, Luoyang, 471003 Henan China; 2 Information Engineering College, Henan University of Science and Technology, Kaiyuan Avenue, Luoyang, 471003 Henan China

**Keywords:** Expression recognition, Canny edge detection, Depthwise separable convolution, Inverted residual module

## Abstract

A single network model can’t extract more complex and rich effective features. Meanwhile, the network structure is usually huge, and there are many parameters and consume more space resources, etc. Therefore, the combination of multiple network models to extract complementary features has attracted extensive attention. In order to solve the problems existing in the prior art that the network model can’t extract high spatial depth features, redundant network structure parameters, and weak generalization ability, this paper adopts two models of Xception module and inverted residual structure to build the neural network. Based on this, a face expression recognition method based on improved depthwise separable convolutional network is proposed in the paper. Firstly, Gaussian filtering is performed by Canny operator to remove noise, and combined with two original pixel feature maps to form a three-channel image. Secondly, the inverted residual structure of MobileNetV2 model is introduced into the network structure. Finally, the extracted features are classified by Softmax classifier, and the entire network model uses ReLU6 as the nonlinear activation function. The experimental results show that the recognition rate is 70.76% in Fer2013 dataset (facial expression recognition 2013) and 97.92% in CK+ dataset (extended Cohn Kanade). It can be seen that this method not only effectively mines the deeper and more abstract features of the image, but also prevents network over-fitting and improves the generalization ability.

## Introduction

With the breakthrough and innovation of face recognition technology [45], when it is inconvenient to hold the remote control or touch the screen in life, research has gradually been devoted to the manipulation of virtual reality objects by facial expressions. Therefore, facial expression recognition has great potential application value in virtual reality, intelligent machines and other fields. The specific application scenarios of expression recognition have greatly promoted the development of science and the progress of society. In terms of human-computer interaction [13], it can accompany and care for the empty nesters and left-behind children to a certain extent, alleviate loneliness and make up for emotional deficiency in life; In terms of medical and health care [34], especially in recent years, the COVID-19 has spread at home and abroad. In the treatment of patients, expression recognition can help medical staff analyze the changes of patients’ conditions, and timely find out the patients’ physical discomfort and medication effects. This can effectively reduce the workload and work pressure of doctors and nurses, and reduce the medical cost of treating patients in hospitals; In terms of cloud classroom education [25], due to the serious impact of the epidemic, students’ online classes at home are becoming more and more common. The development of expression recognition technology can provide real-time feedback on students’ listening status and mastery of course content. Teachers can remind students with inattentive attention to listen attentively according to the feedback results, and timely adjust the teaching methods and progress according to students’ mastery of the course, so as to improve classroom efficiency.

Facial expression is the most natural, powerful and universal form of nonverbal communication to convey human emotional state [8, 40]. Emotion recognition [44] is the detailed processing of various signals of human physiology, psychology, expression, behavior, etc. by computer, so as to analyze the real emotional state of human heart. Human beings can recognize the colorful world through body organs such as vision, taste, hearing, smell and touch, while the recognition of human emotional state is largely completed by the mutual assistance of vision and hearing, including timbre, tone, facial expression, text and gestures. Expert A. Mehrabian [24] found through research that in face-to-face conversations between humans, facial expressions [43], language, tones, different postures, etc. can convey communication information, but the information conveyed by expressions accounts for 55% of the proportion, while only 7% depends on the content of the speaker [14]. Therefore, it can be inferred that facial expressions play an irreplaceable role in capturing information during face-to-face conversation, and can make communicators to perceive the other party’s inner feelings and intentions in time.

In 1971, psychologist Ekman et al. [9] made great contributions to the development of facial expression recognition. They first proposed that there would be six basic emotions on the face, which could truly reflect people’s psychological state, and divided the emotions into six basic expressions of happiness, surprise, fear, sadness, anger and disgust according to their different characteristics. At the same time, the facial action coding system is also constructed in detail to express the features of each expression, such as eyebrows naturally raised upwards or eyebrows tightly wrinkled in the middle; Small fine lines appear around the eyes, the facial muscles stretch upward; The jaw drops, the mouth is slightly opened and stretched outward, the upper and lower lips are separated, but the mouth muscles are not tense or stretched, etc. Because a single traditional feature extraction algorithm can’t extract high-level features, which leads to low recognition accuracy, researchers from all walks of life try to use different methods to carry out facial expression recognition. By applying deep neural network to experimental research, deeper features are extracted to improve the accuracy of facial expression recognition. In the 1990s, with the development of the practical application of pattern recognition technology and the continuous improvement of image processing technology, it is gradually possible for computer to analyze and process expression feature classification independently. In 2006, the National Natural Science Foundation of China officially approved the related research on facial expression recognition, which promoted the development of domestic facial expression recognition research. Facial expression recognition has gradually developed in recent years and has entered our society and life. Due to the diverse and complex characteristics of facial expressions, and involves two aspects of human physiology and psychology, it has increased a lot of difficulties for facial expression recognition. He et al. [15] proposed a multi-angle facial expression recognition method that uses PCA to select the incremental correction features of the regression model, and verified that the proposed method is better than the traditional method. At the same time, the facial skin state also affects the result of expression recognition [3]. Gao et al. [10] proposed a three-channel facial expression recognition method focusing on the face, eyes and mouth regions, which improved the recognition rate of expression as a whole. Li et al. [18] proposed a reinforcement learning framework consisting of image selector and rough emotion classifier, in which the image selector selects useful images for emotion classification through reinforcement strategy, and the rough emotion classifier is used to train the image selector. The framework improves the classification performance by improving the quality of images. According to the problem of poor recognition effect caused by face occlusion in real life, Li et al. [19] proposed a convolutional neural network with attention mechanism (ACNN), which can perceive the occluded area of the face and focus on the most discriminative non-occluded area. The network improves the recognition accuracy of non-occluded faces and occluded faces. With the development of facial expression recognition research, the research results are gradually applied to remote network monitoring. Teachers can see the attendance situation every day by detecting faces and judge whether students listen carefully in class through facial expressions [17]. Transfer learning is gradually widely used in deep learning. When constructing network models, transfer learning can reduce the negative transfer of facial expressions and capture better facial expression features [26]. In [11], the author proposed an occluded expression recognition model based on the generated countermeasure network, which includes two modules: occluded face image restoration and face recognition. In [36], the author proposed an efficient dual integrated convolutional neural network (DICNN) model, which is fine-tuned on the FER2013 dataset to effectively improve the accuracy of expression recognition. The research status of facial expression is shown in Table 1.

**Table 1:** Research status of facial expression recognition

Researcher	Method
Ekman et al.	They first proposed that there would be six basic emotions on the face.
He et al.	They proposed a multi-angle facial expression recognition method that uses PCA to select the incremental correction features of the regression model.
Gao et al.	They proposed a three-channel facial expression recognition method focusing on the face, eyes and mouth regions.
Li et al.	They proposed a reinforcement learning framework consisting of image selector and rough emotion classifier
Li et al.	They proposed a convolutional neural network with attention mechanism, which can perceive the occluded area of the face and focus on the most discriminative non-occluded area.
Minoofam et al.	The author uses facial detection through Haarcascades and facial recognition using feature-based methods, namely - PCA and LDA.
Ge et al.	The author proposed an occluded expression recognition model based on the generated countermeasure network, which includes two modules: occluded face image restoration and face recognition.
Saurav et al.	The author proposed an efficient dual integrated convolutional neural network (DICNN) model, which is fine-tuned on the FER2013 dataset to effectively improve the accuracy of expression recognition.
Ours	We proposes a facial expression recognition method based on the improved depthwise separable convolutional network. Firstly, Gaussian filtering is performed by Canny operator to remove noise, and combined with two original pixel feature maps to form a three-channel image. Then, the network model is constructed by combining the Xception model and the inverted residual structure.

In the deep neural network, a large amount of data is trained by establishing a model with a multi-layer structure, so as to obtain effective features and improve the accuracy of recognition. In order to solve the problems that a single network model cannot extract more complex and rich effective features, and the network structure is huge, there are many parameters and consume more space resources, this paper proposes a facial expression recognition method based on the improved Xception network model. This method mines the deeper and more abstract features of the image, and reduces the influence of illumination, posture, etc. In this paper, the Canny operator is used for convolution denoising, which can reduce the scale of image features while retaining the original image features, which can effectively remove image noise and find the strong edges of the image. The Xception network model was an optimization and improvement of Inception-v3 by Google after Inception network architecture was proposed. It uses depthwise convolution and pointwise convolution to extract more effective features through different channels and different convolution kernels, which greatly reduces the number of parameters and computation cost. The inverted residual structure in the MobileNetV2 network is introduced into the improved Xception network structure, which solves the problems of gradient disappearance and gradient explosion, and improves the propagation ability of gradient between product layers. The amount of data available at home and abroad is limited, and it is easy to cause over-fitting when the neural network model is trained in the experiment. Therefore, this paper adopts the data enhancement method to expand the image sample size. However, the model built in this paper also has some shortcomings, such as low recognition rate for images with large noise, low resolution and serious occlusion. In future work, we will continue to study these problems.

## Edge detection

**Fig. 1:**
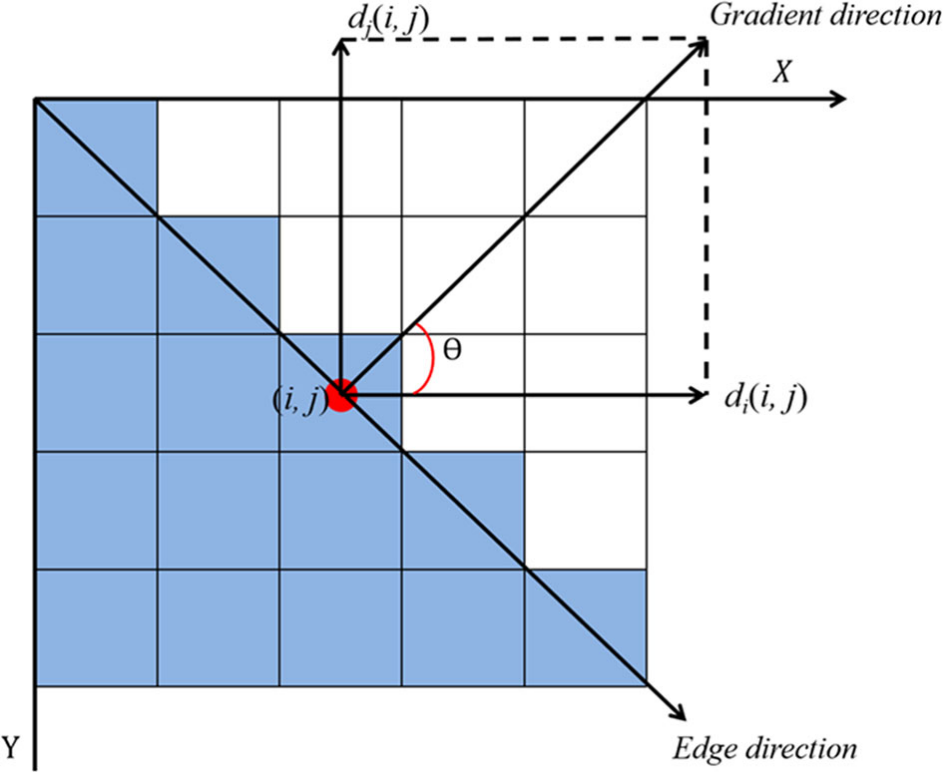
Schematic diagram of gradient vector and edge direction

**Fig. 2:**
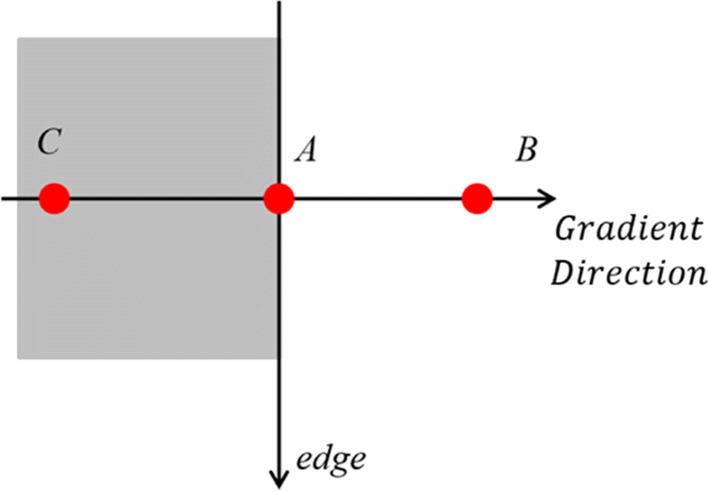
Schematic diagram of non-maximum suppression

**Fig. 3:**
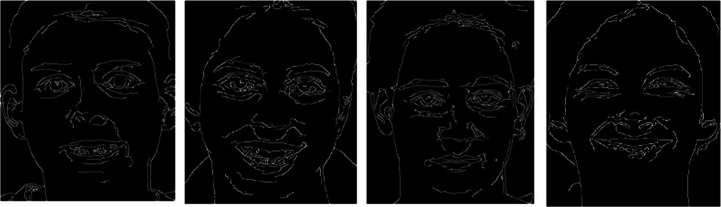
Edge detection diagram. The network structure constructed in this paper is the input corresponding to the shape of the three-channel image. The input image is processed by the Canny operator to remove noise (The feature map extracted by Canny operator is marked as *m*), and combined with the two original pixel feature maps (The two original pixel feature maps are marked as *n*_1_, *n*_2_ respectively) to form a three-channel image (*M* = [*m*,*n*_1_,*n*_2_]). Then the three-channel image is input into the constructed network for training experiments. This method of combining three-channel feature maps reduces the loss of effective features and improves the recognition accuracy

Canny edge detection was proposed in 1986 and has the characteristics of filtering and enhancement, which can effectively remove noise and extract edge features. It has achieved good results in image processing in the field of image recognition. Compared with Sobel, Prewitt and other operators, Canny edge detection can further refine and locate edges more effectively and accurately [22]. The detailed steps of Canny operator to extract features are as follows:

Step 1: Image noise reduction. Gaussian filtering is applied to the input image to remove noise and reduce the recognition rate of false edges [41].
  1 $$ h(x, y)=\frac{1}{2 \pi \sigma^{2}} e^{-\frac{x^{2}+y^{2}}{2 \sigma^{2}}} $$
  where (*x*,*y*) is the vertical and horizontal coordinate points, *σ* is the standard deviation of Gaussian function, ∗ represents convolution, and *g*(*x*,*y*) is the smoothed image, which can be expressed as *g*(*x*,*y*) = *h*(*x*,*y*) ∗ *f*(*x*,*y*), and the size of Gaussian filtering kernel is (5,5).
Step 2: Find the edges. The amplitude and direction of the gradient are calculated by the finite difference of the first-order partial derivative. The first-order partial derivative matrices in the x and y directions are *P*(*i*,*j*) and *Q*(*i*,*j*):
  2 $$ \left\{\begin{array}{l} P[i, j]=(f[i+1, j]-f[i, j]+f[i+1, j+1]-f[i, j+1]) / 2 \\ Q[i, j]=(f[i, j]-f[i, j+1]+f[i+1, j]-f[i+1, j+1]) / 2 \end{array}\right. $$
  The gradient amplitude *M*(*i*,*j*) and the gradient direction *𝜃*(*i*,*j*) are calculated from (2):
  3 $$ \left\{\begin{array}{l} M[i, j]=\sqrt{P[i, j]^{2}+Q[i, j]^{2}} \\ \theta[i, j]=\arctan (Q[i, j] / P[i, j]) \end{array}\right. $$
  where *P* represents the gradient amplitude in *x* direction, *Q* represents the gradient amplitude in y direction, and *M*(*i*,*j*) reflects the edge intensity of the image, *𝜃*(*i*,*j*) reflects the edge direction, as shown in Fig. 1.
Step 3: Refine the edges. At each point, the center pixel *A*(*x*,*y*) of the field is compared with the two pixels along its corresponding gradient direction, if the center pixel is the maximum value, it will be retained; Otherwise, *A*(*x*,*y*) = 0, which can suppress the non-maximum value and retain the point with the maximum local gradient to obtain the refined edges, as shown in Fig. 2.
Step 4: Detect and connect edges with double threshold algorithm. The selected high threshold and low threshold are 150 and 50 respectively, and the points less than the low threshold was set to 0; The points greater than the high threshold was set to 1; The points less than the high threshold but greater than the low threshold was determined using 8-connected regions. The edge detection Canny feature map extracted in this paper is shown in Fig. 3.

## Construction of the neural network model

### Lightweight neural network architecture

**Fig. 4:**
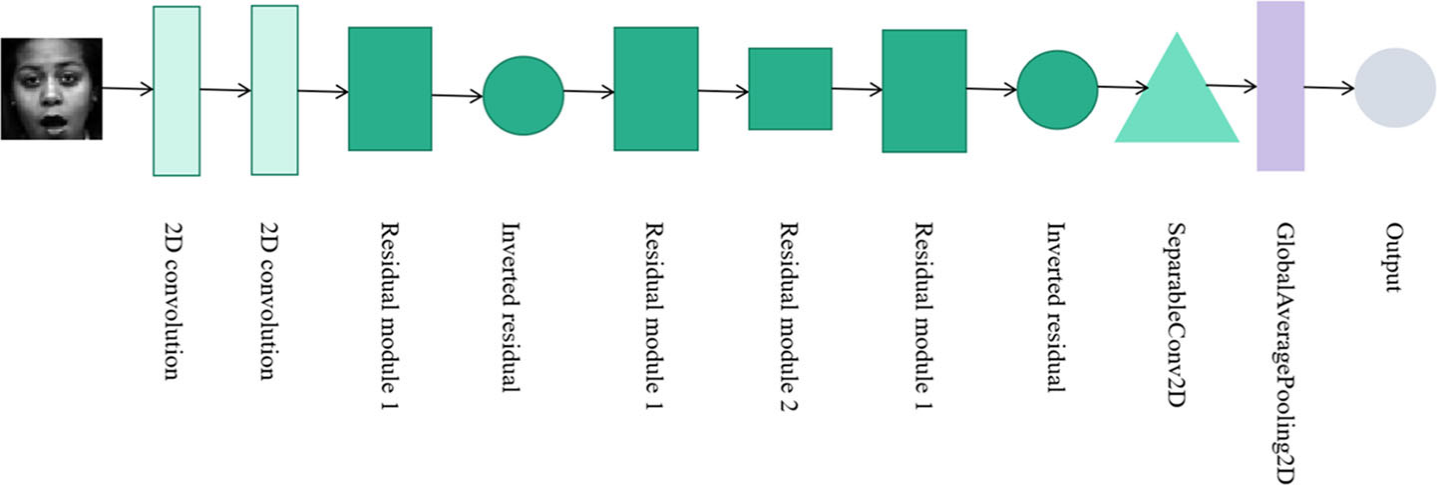
Neural network architecture diagram. Each graph represents a network module, and different graphs represent different network modules. Image data is input from the left, and features are extracted through the middle layer in turn, and finally the classification results are output through the Softmax function

In order to reduce the number of network weights, reduce the complexity of the network model structure and improve the network accuracy, the network model in this paper reduces the amount of network parameters by partial connection and weight sharing, and the constructed network structure has the characteristics of hierarchical expression. At the same time, the ideological architecture of depthwise separable convolution is adopted. In the Xception network module, 1 × 1 convolution operation is performed firstly on the input image, and then 3 × 3 convolution operation is performed on each channel after the convolution, and finally the results are connected together. Compared with the Inception-v3 [38] network model, it improves the effect of network model without increasing network complexity. In this paper, the residual connection mechanism is used in the constructed network model to solve the problems of network performance degradation and gradient disappearance, so that we can train deeper network while ensuring good performance. The structural design diagram of lightweight neural network is shown in Fig. 4:

In the feature extraction operation, the convolution calculation is a process in which the convolution kernel moves on the feature map according to the set convolution step size, and the pixel value is multiplied and summed with the convolution kernel. The convolution operation is a basic step in the network structure, and its main function is to extract the effective features of the image by enhancing the original signal features of the image and reducing noise [5]. In the process of convolution, in order to solve the problem of image reduction and loss of most of the information on the edge of the image every time the convolution operation is performed, it is necessary to select different convolution operation padding according to the constructed network model, in which the Valid convolution method is no padding without any modification, and the Same convolution method is natural padding. The calculation process of the convolution operation is as follows:

4 $$ {X_{j}^{L}}=g\left( \sum\limits_{i} X_{i}^{L-1} \theta_{i j}+b\right) $$

where ${X_{j}^{L}}$ is the *j*-th feature map unit of the *L*-th layer, $X_{i}^{L-1}$ is the *i*-th input of the *L* − 1-th layer, *𝜃*_*ij*_ represents the convolution kernel, *b* is the bias unit, and *g*(*x*) is the activation function.

The network model adopts the maximum pooling of spatial data behind the depthwise separable convolution layer, which is used for feature compression to extract the main features and simplify the network complexity, realizing feature dimension reduction and effectively preventing over-fitting [44]. The common method is to select the maximum or average value of the local area.

**Fig. 5:**
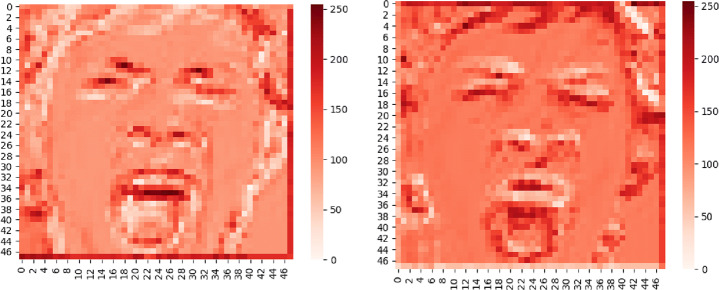
Thermodynamic diagram of facial expression feature

In this paper, global average pooling is used to replace the fully connected layer, which can not only reduce the number of parameters to prevent over-fitting [20], but also sum up the spatial information, so that the spatial transformation of input is more stable. The thermodynamic diagram of facial expression features in the network structure of this paper is shown in Fig. 5.

### Depthwise separable convolution

**Fig. 6:**
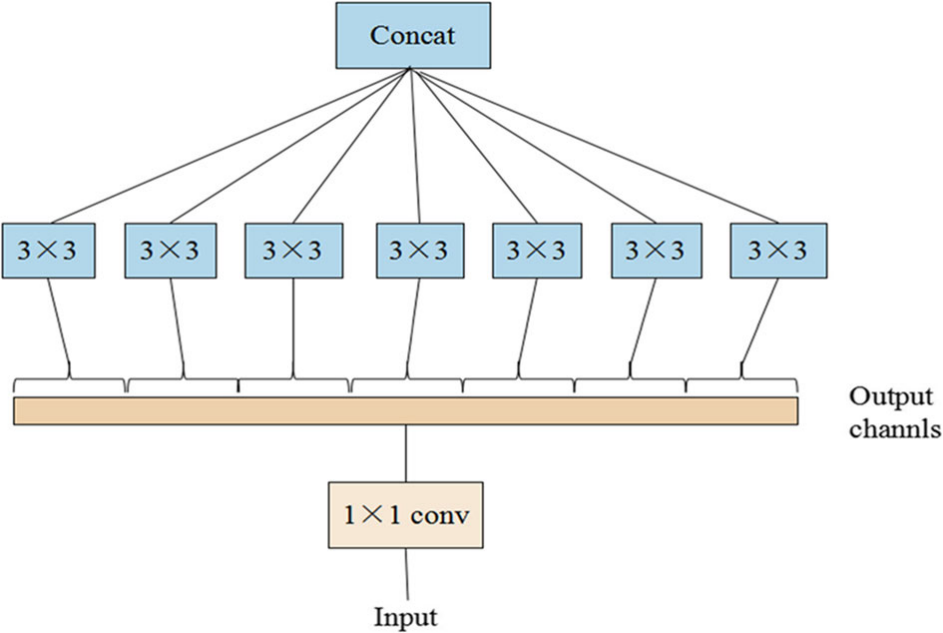
Cross-channel correlation and spatial correlation decoupling diagram. First, 1 × 1 channel correlation convolution is performed, and then the number of 3 × 3 convolution is the same as the number of output channels of 1 × 1 convolution

In order to reduce the number of network parameters and improve the performance of the constructed network model, we construct the network model by separately processing the channel correlation and the spatial correlation. The network structure of the depthwise separable convolution method we use to extract image sample features is to perform the pointwise convolution operation in the first step, and then perform the depthwise convolution operation in the second step. The two convolution operations have different functions in extracting features in different channels, and the convolution kernel for the convolution operation is also different. In order to ensure that the data is not destroyed, there is no nonlinearity caused by Relu between the two convolution operations, as shown in Fig. 6. Compared with standard convolution, this convolution operation method of extracting features in two steps reduces the space cost of constructing network model and the time cost of training network model, and also achieves the effect of extracting image features by the standard convolution operation, which greatly reduces the amount of calculation while maintaining the accuracy of the neural network.

### Activation function

In order to solve the problem of insufficient expression ability of linear model in gradient backpropagation in deep neural network. This paper uses ReLU6 mentioned in MobileNetV2 as the nonlinear activation function. ReLU6 is to limit the maximum output value to 6 in the Relu function, which not only speeds up the convergence speed of gradient descent at low precision, but also improves the sparse expression ability of the neural network [31, 32]. The ReLU6 function is defined as follows:

5 $$ R \mathrm{e} L U 6=\min \{\max (0, x), 6\} $$

where, *x* is the output feature of the previous network layer.

### Residual network

**Fig. 7:**
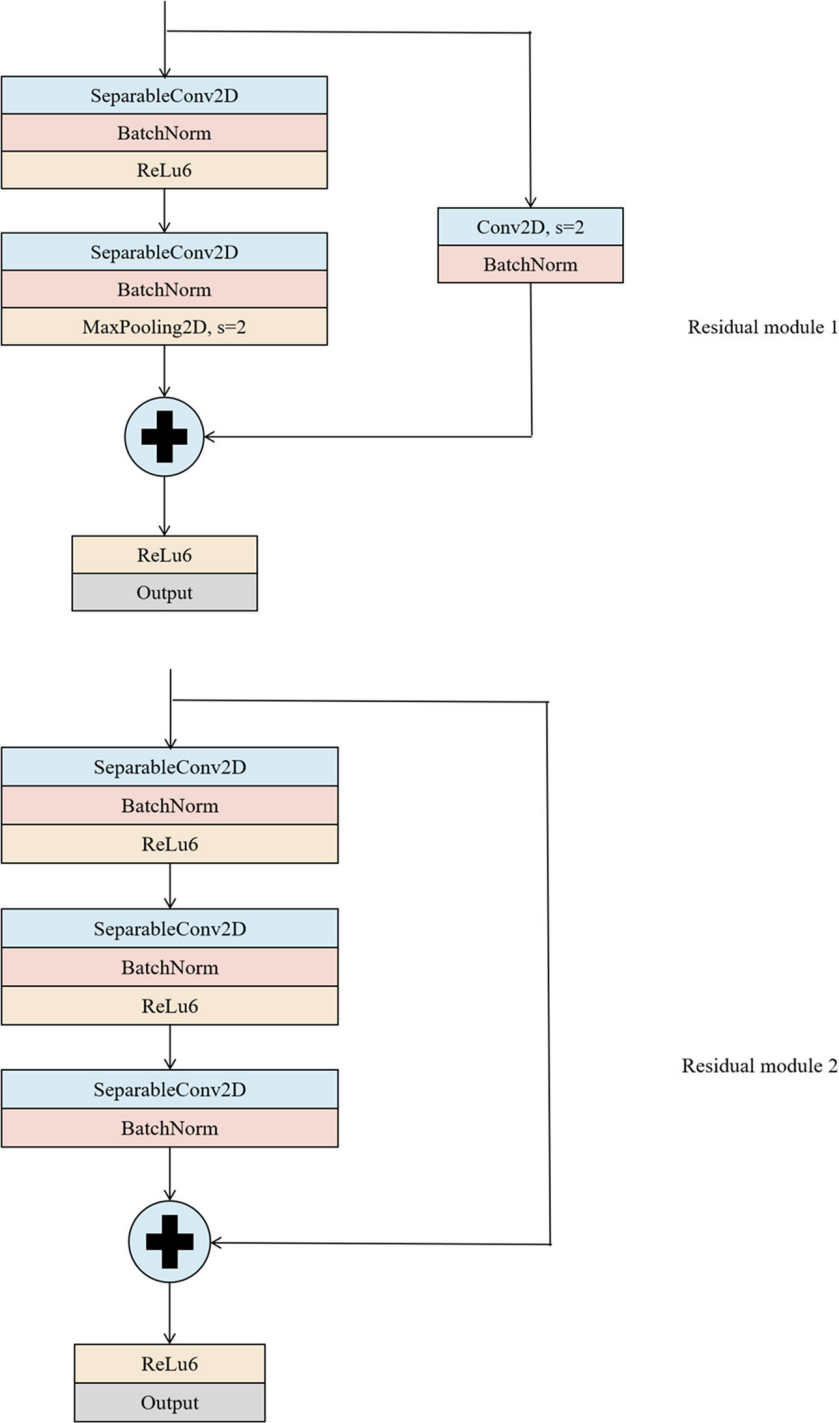
Two residual network structure diagrams. All 2D Convolution and Separable Convolution layers are followed by batch normalization. All network layers use the Relu6 activation function

In the process of constructing the network structure, with the increase of the number of hidden layers in the network and the deepening of the network, the volume of the network model will become larger, the number of parameters will increase, and the self-learning efficiency will be reduced. At the same time, it will also affect the results of image feature classification. In order to solve the above problems that may be caused by the deep network structure, this paper uses the residual connection mechanism in Resnet [6] to construct the network model, and changes the convolutional layer into a convolution mode in which pointwise convolution operation is performed in the first step and depthwise convolution operation is performed in the second step. The residual structure can train deeper neural network, and can solve the problems of network performance degradation and gradient disappearance caused by depth deepening. The introduction of residual structure in deep neural network not only significantly speeds up the convergence process of the network but also achieves higher accuracy. The residual network structure is shown in Fig. 7.

### Inverted residual module

**Fig. 8:**
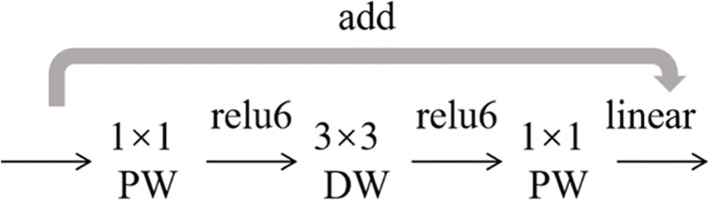
Inverted residual microstructure

When depthwise convolution extracts features, it filters spatial information independently channel by channel, the convolution kernels corresponding to different channels are also different, and the resulting feature maps are unrelated to each other, and the number of feature maps is exactly the same as the number of input channels. Therefore, the step of depthwise convolution cannot change the number of channels. The number of channels of the previous layer determines whether the features extracted by depthwise convolution are in low-dimensional or high-dimensional space. If it is in low-dimensional space, it will result in the inability to extract complete features. In the inverted residual structure adopted in this paper, a layer of 1 × 1 convolution is added before each depthwise convolution, which aims to improve the dimension and obtain more features. In this paper, the expansion factor *t* = 6 is applied to the size of the input tensor, which can solve the problem caused by the small number of channels. After the pointwise convolution (PW) operation, the dimension can be improved, so that the second step performs the depthwise convolution (DW) operation to extract features in the high-dimensional space, as shown in Fig. 8. Firstly, map the image into high-dimensional space through 1 × 1 convolution and use depthwise convolution to extract features, and then use 1 × 1 convolution to map the features back to the low-dimensional space [35]. Since the nonlinear activation function will destroy the features in the low-dimensional space, so the activation function is not added after the last 1 × 1 convolution. This network structure can improve the ability of information dissemination and prevent the problem of gradient disappearance during gradient backpropagation [43].

### Network architecture

This paper proposes a facial expression recognition method based on the improved depthwise separable convolutional network. The three-channel image composed of the feature map extracted by Canny edge detection and two original pixel feature maps is input into the constructed lightweight neural network. The design of the network adopts the structure recognition features based on Xception [7], which mainly relies on the layer-by-layer processing of the three-channel features after channel fusion to improve the accuracy of facial expression recognition. The proposed network structure and detailed parameter information are shown in Fig. 4 and Table 2. Firstly, the convolution operation is performed through two 2D convolution layers in turn. The size of the convolution kernel is 3 × 3, the number of convolution kernels is 32 and 64 respectively, and the stride is 1; Secondly, the output is sequentially passed through residual module 1, inverted residual microstructure, residual module 1, residual module 2, residual module 1, and inverted residual microstructure in turn. The number of convolution kernels is 128, 256, 256, 364, 364, and 512 respectively, where the convolution kernel size of depthwise separable convolution is 3 × 3, the convolution kernel size of MaxPooling is 3 × 3, the convolution kernel size of 2D convolution layer is 1 × 1. The step size of residual module 1 is 2, and other steps are 1; Thirdly, the output is sent to the depthwise separable convolution layer in turn, the size of the convolution kernel is 3 × 3, the number of convolution kernels is 728, and the step size is 1; Finally, the output is sent to the global average pooling layer and Softmax classifier. All 2D convolution and depthwise separable convolution operations must go through a Batch Normalization layer and ReLU6 activation layer to accelerate the convergence speed of the constructed network structure and increase the ability to extract nonlinear features.

**Table 2:** Network structure and parameter table

Input	Layer	*c*	*s*
48 × 48 × 3	Conv2D	32	1
48 × 48 × 32	Conv2D	64	1
48 × 48 × 64	Residual module 1	128	2
24 × 24 × 128	Inverted residual structure × 4	256	1
24 × 24 × 256	Residual module 1	256	2
12 × 12 × 256	Residual module 2	364	1
12 × 12 × 364	Residual module 1	364	2
6 × 6 × 364	Inverted residual structure × 2	512	1
6 × 6 × 512	SeparableConv2D	728	1
6 × 6 × 728	GlobalAveragePooling2D	−	−
1 × 1 × 728	Conv2D	7	−

## Experimental results and analysis

### Experimental data and platform

This paper focuses on the research of facial expression recognition for static images. However, in practical applications, it is often the video recorded by the camera or the real-time monitoring video. Therefore, the method in this paper is insufficient for facial expression recognition of video. In this paper, the two datasets we choose are Fer2013 [12] and CK+ [21]. In the process of training the network model, the image sample size is expanded by data enhancement method, which improves the performance of the constructed network structure and prevents the over-fitting of the network model. The datasets adopt 10 fold cross validation method to divide the image data of Fer2013 and CK+ datasets, divides the expression images into ten parts, trains the network model in a loop. Each time, one different part is used to test the results, and the rest is used to train the model, and the average crossover experiment accuracy of 10 times is taken as the result [29, 42]. The experimental software platform is Python 3.7 under Linux, using the Keras framework with TensorFlow as the backend. The hardware platform is Dell Poweredge R940xa, and the GPU is a 16GB NVIDIA Tesla T4. We use the batch size of 64, the initial learning rate of 0.001, Adam optimizer to optimize the training process, and the width multiplier *α* of 1 in the network, *α* can reduce the number of network parameters, which has a better effect than reducing the depth of the model.

#### 1) Fer2013 dataset

**Fig. 9:**
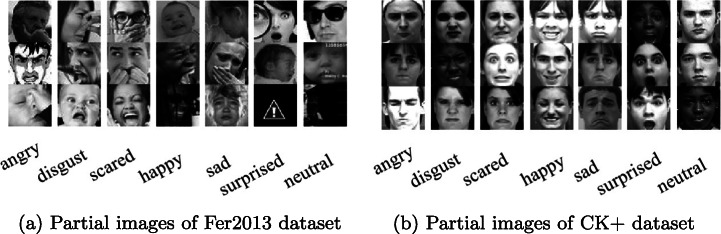
Figures (a) and (b) are the original pictures in the two datasets. Figure (a) was taken in a natural environment with many interference factors, and Figure (b) was taken in a laboratory environment with relatively few interference factors

Fer2013 is an expression image dataset for Kaggle competitions, and it is also an open international dataset. The image data in Fer2013 was collected through Google search, and the face area in the image was collected using OpenCV face recognition [27, 46]. This dataset does not directly show the image, but saves the expression category, pixel value, and purpose into a CSV file. This paper focuses on the Fer2013 dataset. The expression images are collected in the living environment. The image quality and resolution are not high [2], and they are affected by light intensity, different races, different skin colors, and various degrees of occlusion, which are closer to real life scenes. At the same time in the dataset, there are facial expressions of children and the elderly, as well as facial appearances like cosmetic and aging [4]. The dataset contains 4953 “angry” images, 547 “disgust” images, 5121 “scared” images, 8989 “happy” images, 6077 “sad” images, 4002 “surprised” images, and 6198 “neutral” images, with a total of 7 expressions. Figure 9(a) shows some samples from the Fer2013 expression dataset.

#### 2) CK+ dataset

The images in the CK+ dataset were taken in the laboratory environment and consisted of 593 expression sequences of 123 people. For each image sequence, the expression slowly changed from neutral to peak. Different sequences were marked with different codes, and the expression codes ranged from 0 to 7. There are 6 basic expressions in the CK+ dataset plus contempt and neutral. This paper selects 7 expressions of angry, neutral, disgust, scared, happy, sad, and surprised, as shown in Fig. 9(b).

### Analysis of experimental results

**Fig. 10:**
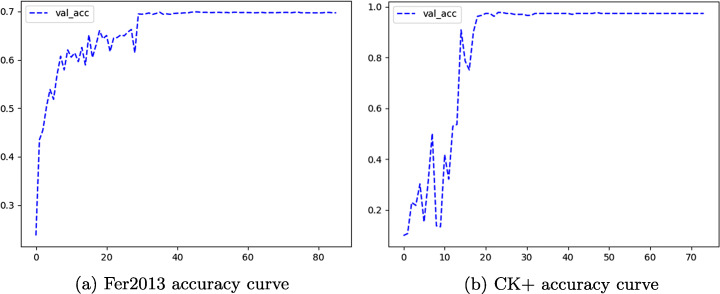
Figures (a) and (b) are the accuracy curves of two datasets. The abscissa represents the training rounds and the ordinate represents the accuracy of the corresponding rounds

In order to train a network model with stronger robustness and better generalization ability, this paper optimizes the training model for many times. The accuracy iterative convergence curves on Fer2013 and CK+ datasets are drawn in Fig. 10(a) and (b) respectively. In the Fer2013 dataset, the accuracy of human recognition is 65%± 5%. The model fluctuates greatly in the iteration rounds from 10 to 30. On the whole, the accuracy rate gradually increases and stabilizes with the iteration rounds, and the highest accuracy rate reaches 70.76%, which exceeds the effect of human recognition. The experimental result is 2% higher than the single model MobileNetV2 and 2.6% higher than Xception. In the CK+ dataset, the accuracy fluctuates greatly before the iteration rounds are 16, and finally tended to a stable and convergent state, with the highest accuracy rate reaching 97.92%. In [33], the constructed CNN model was proposed, and the CK+ dataset weight is initialized to gaussian+msra. The experimental results showed that the accuracy of expression recognition is 93.15%.

**Fig. 11:**
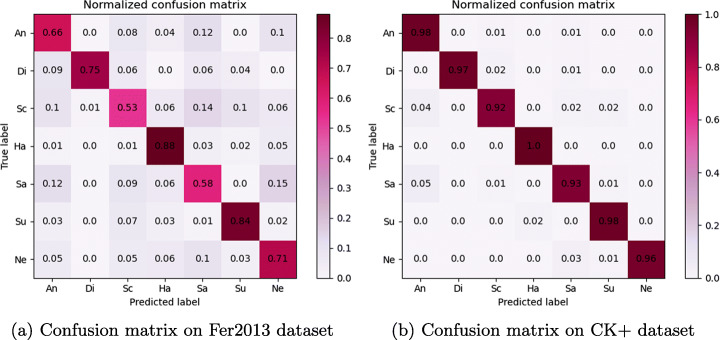
Figures (a) and (b) are the confusion matrices of the two datasets, where An, Di, Sc, Ha, Sa, Su and Ne are the first two English letters of the seven expressions, respectively

The experimental results are obtained by constructing a lightweight neural network for expression recognition training. The more image data, the more stable the trained model structure and the stronger the robustness. In this paper, the data enhancement method is used to expand the sample size of the dataset, which effectively improves the generalization ability of the constructed network structure on other datasets. As can be seen from Fig. 11(a) and (b), the confusion matrix of Fer2013 and CK+ datasets shows that the recognition rate of fear and sadness is low. The reason is that Fer2013 dataset has cartoon face images, face images of different ages and skin colors, non-face images, various degrees of occlusion and other interference, which increases the difficulty of feature extraction in the network structure. The possible reason for the CK+ dataset is that the features of scared and sad are similar to some extent, which interferes with the differentiation of different expressions and leads to low accuracy of expression recognition.

### Comparative experiments of different methods

After comparing with other network models, it is verified that the method of combining the Xception module and the inverted residual structure to build a network model improves the accuracy of expression recognition and the robustness of the constructed network to a certain extent. On the Fer2013 and CK+ datasets, this experiment is compared with the expression recognition algorithms of Xception [7], CNN [28], LBP [16, 30], Inception-V4 [37, 39], Canny [1, 23], and MobileNetV2 [35]. The comparison results are shown in Tables 3 and 4. According to the analysis results, compared with the single model, this paper adopts the method of combining multiple models to construct the network structure, which not only reduces the number of parameters to prevent over-fitting, but also solves the problems of gradient disappearance and gradient explosion.

**Table 3:** Recognition results of different methods on the Fer2013 dataset

Methods	*α*	Fer2013 Accuracy
Xception	−	68.12%
CNN	−	66.92%
MobileNetV2	1	68.73%
MobileNetV2	1.4	69.24%
LBP	−	67.89%
MobileNetV2+Canny	1	69.52%
MobileNetV2+Canny	1.4	69.14%
Canny	−	67.42%
Paper method	1.4	69.78%
Paper method	1	70.76%

**Table 4:** Recognition results of different methods on the CK+ dataset

Methods	*α*	CK+ Accuracy
Xception	−	87.24%
Canny	−	86.45%
InceptionV4	−	85.92%
MobileNetV2	1	90.12%
MobileNetV2	1.4	92.75%
MobileNetV2+Canny	1	95.09%
MobileNetV2+Canny	1.4	96.11%
Xception+Canny	−	94.28%
Paper method	1.4	96.04%
Paper method	1	97.92%

## Conclusion

The research of this paper mainly focuses on static pictures. In order to solve the problems that traditional facial expression recognition algorithms can’t extract high-level depth features and the single deep network model has many parameters and weak generalization ability, this paper proposes a facial expression recognition method based on improved depthwise separable convolutional network. Through the combination of traditional feature extraction method and deep neural network, the proposed method effectively mines the deeper and more abstract features of the image, and reduces the influence of illumination, posture, etc. At the same time, the network model is constructed by combining the Xception model and the inverted residual structure, which not only effectively prevents over-fitting but also solves the problems of gradient disappearance and gradient explosion. Experiments show that the model proposed in this paper can effectively improve the accuracy of expression recognition and enhance the robustness and generalization ability of the network. Although the proposed model has achieved a good recognition effect, it is still necessary to further improve the accuracy of the proposed method, which may be affected by various occlusion and posture changes, for obtain better recognition effect.

The expression classification of the current research is based on 7 emotions, but there are often flash expressions on the face, that is, micro-expressions. In the next step, expression recognition based on complex micro-expression features will be the focus of research. To solve the problem of limited image samples when training the model, the next step is to expand the number of images in the way of Generative Adversarial Networks (GAN), and generate image samples with classification labels by learning from the input dataset. This method can prevent over-fitting of the network model for the constructed depth network structure.
